# Cost-effectiveness of dolutegravir vs. efavirenz-based combined antiretroviral therapies in HIV-infected treatment-naive patients in a Nigerian treatment centre

**DOI:** 10.4314/ahs.v23i1.18

**Published:** 2023-03

**Authors:** Abdulmuminu Isah, Patrick O Chukwu, Abubakar Abba, Nneka U Igboeli, Ayogu Ebere, Olusegun F Omotola, Faith A Alozie, Obinna I Ekwunife, Maxwell O Adibe

**Affiliations:** 1 University of Nigeria Nsukka, Department of Clinical Pharmacy and Pharmacy Management; 2 University of Nigeria Teaching Hospital, Department of Pharmaceutical Services; 3 National Assembly, Department of Pharmacy; 4 Nnamdi Azikiwe University, Department of Pharmacy and Pharmacy Management

**Keywords:** Average cost-effectiveness ratio, cost-effectiveness, Dolutegravir

## Abstract

**Background:**

Dolutegravir (DTG) based antiretroviral therapy (ART) has largely replaced Efavirenz (EFV) based therapy as the preferred first-line regimen in the treatment of adults with HIV. This study was carried out to evaluate the comparative cost-effectiveness of DTG and EFV-based ART in HIV-infected treatment-naïve patients in a treatment centre in Nigeria.

**Methods:**

This was a retrospective case-control study of patients initiated on DTG vs. EFV-based regimens from January 2018 to December 2019 at the APIN/HAVARD clinic of Nigeria's Jos University Teaching Hospital. The current viral load result was used to determine treatment effectiveness using a benchmark of ≤200 copies/mL. Sensitivity analysis was carried out to ensure the robustness of the benchmark. The total cost of treatment was obtained by summing up the relevant cost components. Appropriate descriptive and inferential statistics were employed in data analysis using Statistical Product and Services Solutions (SPSS) V.25. The incremental cost-effectiveness ratio of DTG compared to EFV was presented as cost/effectiveness.

**Results:**

Treatment was effective in 42(51.9%) and 58(71.6%) patients initiated on DTG and EFV-based regimen, respectively. The incremental cost-effective ratio (ICER) of patients on DTG compared to those on EFV was $10.5076 per effectiveness, which was less than 1% of the Nigerian 2019 per capita Gross Domestic Product. Sensitivity analysis showed the robustness of the result.

**Conclusion:**

Efavirenz based regimen had higher treatment effectiveness than DTG-based regimen in treatment-naive patients after initiating treatment in a short term. Compared to EFV, DTG-based regimen is cost-effective in the management of treatment naïve HIV patients.

## Background

Nigeria has the fourth-largest HIV epidemic in the world (after South Africa, India and Mozambique), with about two million people living with the infection in 2021^1^,^2^. Although there was a significant decrease in the number of new infections from 2010 to 2014, Nigeria still accounted for more than two-thirds of new HIV infections in sub-Saharan Africa in 2019. HIV also remains an important cause of mortality, with approximately 45,000 HIV related deaths in 2019^2^.

The introduction of highly active antiretroviral therapy (HAART) which is a regimen containing three or more antiretroviral drugs has resulted in a dramatic reduction in morbidity and mortality among the HIV positive population^3^–^5^, with Nigeria not being an exception. Despite the successes achieved with HAART, there were challenges to its use, ranging from high pill burden, numerous drug interaction, and treatment-limiting toxicities 3,6, that prompted the continuous modification of the antiretroviral therapy over the years.

The most recent modification to antiretroviral therapy was the recommendation by the World Health Organization (WHO) for the use of Dolutegravir (DTG) based treatment as the preferred first-line regimen for people living with HIV initiating antiretroviral therapy 7. DTG-based regimens have been reported to exhibit higher efficacy than Efavirenz (EFV) based regimen for treatment-naïve adults 8 . DTG-based regimen also exhibited a more rapid and sustained rate of viral suppression and have a higher genetic barrier for the development of drug resistance when compared with EFV-based regimens 9,10. Nigeria has largely replaced EFV with DTG-based combination therapy as the preferred first-line regimen in treatment-naïve adults and has transitioned those previously on other first-line antiretroviral agents to DTG 11.

Despite the demonstrated higher clinical efficacy of DTG over EFV-based regimen, the economic evaluation of its use in developed countries showed conflicting results. In a study that compared the long-term cost-effectiveness of DTG versus EFV-based regimen from the United States payers' perspective, it was shown that DTG-based regimen was not cost-effective when compared to EFV-based regimen 12. DTG-based combination therapy was however found to be cost-effective for treatment naïve patients in China 13. WHO defines cost-effectiveness analysis as a method that ‘quantifies the gains, or setbacks, in population health as a result of a particular policy or intervention 14. Its goal is to determine the health and environment interventions that should be prioritized in the allocation of resources.

About 65% of all patients living with HIV in Nigeria are currently accessing antiretroviral therapy at no cost^1^. Funding for Nigeria's HIV/AIDS programmes comes mainly from foreign donors and the Nigerian government^15^. Because of the large sum of money spent annually on HIV treatment, the cost-effectiveness of HIV treatment becomes a key consideration to decision-makers. Studies on the cost-effectiveness of DTG-based regimen in Nigeria and other low and middle-income countries are, however, limited. This study was therefore carried out to evaluate the comparative cost-effectiveness of Dolutegravir and Efavirenz based antiretroviral therapies in HIV-infected treatment-naïve patients in a treatment center in Nigeria.

## Methods

### Study Design

This was a retrospective case-control study involving the use of the AIDS Prevention Initiative in Nigeria (APIN) databases of HIV positive, treatment-naive patients, who received care from Jos University Teaching Hospital (JUTH), Jos, Nigeria, from January 2018 to December 2019. The treatment center at the time of the study manages over 10,000 HIV-infected clients on treatment and care.

### Study Sample

The study sample included all newly diagnosed adult HIV patients who were initiated on antiretroviral therapy at the HIV/AIDS treatment center of JUTH during the study period. To be eligible for inclusion in the study, the patient would have been in treatment for at least 90 days. The patient should also have a documented viral load test result after the minimum number of days.

### Sampling Technique

After obtaining the list of eligible patients, a matching of the patients in both arms (case and control) was done using their gender and age category. This was done to avoid selection bias. The final patients that were used for the study had similar gender and age category distribution and a total sample size ratio of 1:1 for the case (DTG-based regimen) and control (EFV-based regimen)

### Data Collection Tool

The data collection tool consisted of three main domains: sociodemographic characteristics, clinical parameters, and cost of treatment. The sociodemographic characteristics section was used to document patients' age, gender, educational level, occupation, and marital status. The second section consisted of the patients' clinical parameters which included the type of antiretroviral drug, first CD4 count test results, and the first viral load test results after commencing ART. The last section of the data collection tool consisted of items related to the cost of treatment.

### Study Procedure

The clinical and sociodemographic data of patients initiated on either DTG or EFV-based regimen during the study period and who satisfied the eligibility criteria, were abstracted from the patients' records.

### Cost of treatment

Costing was done from the payer's perspective. A wholistic cost, including direct and indirect medical costs were considered. The monthly cost of antiretroviral drugs was obtained from the Global Fund's Pooled Procurement Mechanism Reference Pricing for antiretrovirals (ARVs). The cost of laboratory tests (viral load and CD4 count) was obtained from the Virology Laboratory of JUTH which uses the National Health Insurance Scheme cost. The total cost of laboratory tests was obtained by summing the unit costs of laboratory tests each patient had had during the first three months of treatment.

Personnel cost per patient was obtained from the monthly salary of the personnel (Physicians, Nurses, Pharmacists and Medical laboratory Scientists) that attend to patients during their hospital visit. The personnel cost per patient visit was obtained by calculating the proportion of the monthly salary of the personnel that accounts for the time spent attending to a patient during a hospital visit. The individual cost components were summed up to obtain the total cost of treatment for each patient and an average cost was obtained for each of the two regimens. All costs were adjusted to December 2019 prices using the consumer price index and were expressed in United States (US) Dollars at an exchange rate of NGN360 per US Dollar.

### Treatment Effectiveness

The current viral load test result after the initiation of antiretroviral therapy was used as the measure of effectiveness in both DTG and EFV regimen group. Antiretroviral therapy was defined to be effective in patients with a viral load of 200 copies/mL or less using the US Centres for Disease Prevention and Control (CDC) benchmark of viral suppression 16. Effectiveness per regiment was measured as the ratio of patients in whom a given regimen was effective to the total number of patients on that regimen.

### Data Analysis

Data was collected into Microsoft Excel (2016) and checked for completeness. The cleaned data was transferred into Statistical Product and Services Solutions (SPSS) version 25 for analysis. Descriptive statistics were used to analyze the sociodemographic and clinical characteristics of the patients. Chi-square test was used to determine the association between sociodemographic characteristics, clinical characteristics, and the type of ART regimen. An Independent T-test was used to compare the mean clinical characteristics of the patients based on the ARV regimen. The sociodemographic predictors of treatment effectiveness were assessed using multivariate logistic regression.

### Economic Analysis

Cost-effectiveness for each of the regimens was measured as the ratio of the average cost per patient to the average effectiveness per regimen. An incremental cost-effectiveness ratio (ICER) was obtained as the ratio of the cost difference to effectiveness difference of DTG compared to EFV. The incremental cost-effectiveness ratio was reported as cost per effectiveness. A treatment was considered to be cost-effective if its incremental cost per effectiveness was not more than 51% of 2019 Nigerian per capita Gross Domestic Product (GDP) of $2229.859. This assumption was based on the recommendations by Woods et al for the ICE threshold of health interventions in lower-middle-income countries 17,18.

### Sensitivity Analysis

The robustness of the ICER findings was tested using a sensitivity analysis wherein the borders used in classifying the patients' viral load results apart from 200 copies/mL were expanded. The threshold for effectiveness was varied to 150, 100, and 50 copies/ ml and the ICER ratio was measured at these levels of effectiveness.

## Results

### Patients' Sociodemographic Characteristics

A total of 253 patients met the study's eligibility criteria. However, 162 were selected after the age/gender matching to have a 1:1 ratio in total sample size: 81 each in the EFV and DTG groups. Both groups had 46 (56.8%) patients each being males, and 30 (37.0%) patients each being withing 30-39 years age. Most of the patients in both categories were between 30 to 39 years (n=97, 38.3%), and were married (n=126, 50.4%). Only marital status was observed to have a relationship with the antiretroviral category of the patients (p = 0.020). The sociodemographic characteristics are presented in Table 1. All chi-square tests had degree of freedom of 2, as all comparisons were between the two regimens.

**Table 1:** Sociodemographic Characteristics of the Patients based on the Treatment Regimens

Characteristics	Types of Regimen		Total	Chi-square Test	*p*-value

	EFV-Based	DTG-Based			
Gender	n (%)				
Male	46(56.8)	46(56.8)	92(56.8)		
Female	35(43.2)	35(43.2)	70(43.2)	0.000	1.000
Total	81(100.0)	81(100.0)	162(100.0)		
Patients' Age (Years)					
20 – 29	9(11.1)	9(11.1)	18(11.1)	0.000	1.000
30 – 39	30(37.0)	30(37.0)	60(37.0)		
40 – 49	28(34.6)	28(34.6)	56(34.6)		
>50	14(17.3)	14(17.3)	28(17.3)		
Total	81(100.0)	81(100.0)	162(100.0)		
*Mean (SEM)*	*40.28(0.726)*				
Occupation of Patients					
Self Employed	45(56.2)	35(43.2)	80(49.7)	6.155	0.188
Civil Servant	22(27.5)	20(24.7)	42(26.1)		
Unemployed	5(6.2)	12(14.8)	17(10.6)		
Armed Forces	3(3.8)	7(8.6)	10(6.2)		
Student	5(6.2)	7(8.6)	12(7.5)		
Total	80(100.0)	81(100.0)	161(100.0)		
Marital status of patients					
Married	39(48.8)	45(55.6)	84(52.2)	11.683	0.020*
Single	18(22.5)	20(24.7)	38(23.6)		
Separated	12(15.0)	1(1.2)	13(8.1)		
Widowed	10(12.5)	11(13.6)	21(13.0)		
Divorced	1(1.2)	4(4.9)	5(3.1)		
Total	80(100.0)	81(100.0)	161(100.0)		

* Significant at *p* < 0.05

### Clinical Characteristics

Table 2 shows the clinical characteristics of patients in both treatment categories. Majority of the patients in the DTG-based regimen category (n=49, 60.5%) had a disease duration of 181-360 days. For the EFV-based regimen category, majority of the patients (n=40, 49.4%) had a disease duration of 89-180. Also, 49 (60.5%) and 46 (58.2%) % of patients on EFV and DTG respectively had a CD4 count of 200 cells/uL or less. The proportion of patients on EFV and DTG-based regimen who had a viral load of more than 500 copies/mL were 21 (25.9%) and 37 (45.7%) respectively. A comparison of the mean clinical characteristics of the patients (Table 3) showed that those on DTG-based regimen had a higher percentage adherence (p = 0.048) and lower viral load count (p = 0.048) than those on DTG-based regimen.

**Table 2:** Clinical Characteristics of the Patients based on their Treatment Regimens

Characteristics	Types of Regimen		Total	Chi-square Test	*p*-value

	EFV-Based	DTG-Based			
Patients' Duration of Disease(days)	n (%)				
89–180	40(49.4)	31(38.3)	71(43.8)	8.352	0.015*
181–360	34(42.0)	49(60.5)	83(51.2)		
>360	7(8.6)	1(1.2)	8(4.9)		
Total	81(100.0)	81(100.0)	162(100.0)		
*Mean (SEM)*	*210.90(6.643)*				
Patients' Adherence (%)					
<96	23(31.1)	9(12.0)	32(21.5)	8.042	0.005*
96 and above	51(68.9)	66(88.0)	117(78.5)		
Total	74(100.0)	75(100.0)	149(100.0)		
*Mean (SEM)*	*106.80(4.367)*			
Patients' CD4 Count (cell/µL)					
≤200	49(60.5)	46(58.2)	95(59.4)	1.544	0.672
201–500	26(32.1)	28(35.4)	54(33.8)		
501–1000	6(7.4)	4(5.1)	10(6.2)		
>1000	0(0.0)	1(1.3)	1(0.6)		
Total	81(100.0)	79(100.0)	160(100.0)		
*Mean (SEM)*	*217.21(15.450)*				
Patients' Viral Load (copies/mL)					
<201	58(71.6)	42(51.9)	100(61.7)	8.307	0.040*
201–350	1(1.2)	2(2.5)	3(1.9)		
351–500	1(1.2)	0(0.0)	1(0.6)		
>500	21(25.9)	37(45.7)	58(35.8)		
Total	81(100.0)	81(100.0)	162(100.0)		
*Mean (SEM)*	*219530.75(62693.380)*				

* Significant at p < 0.05

**Table 3:** Comparison of Means of Clinical Characteristics of the Patients Based on the Patients' ARV Regimen

Characteristics	Type of ARV	N	Mean	Std. Deviation	Std. Error Mean	F	df	*p*-value
Duration of Disease(days)	EFV-Based	81	204.98	86.894	9.655	0.043	160	0.836
	DTG-Based	81	216.81	82.249	9.139			
Age of Patients(years)	EFV-Based	81	40.06	9.096	1.011	0.134	160	0.715
	DTG-Based	81	40.51	9.424	1.047			
Percentage Adherence (MPR)	EFV-Based	74	97.9730	12.10001	1.40660	3.959	147	0.048*
	DTG-Based	75	1.1551E2	73.36661	8.47165			
CD4 Count of Patients (cells/µL)	EFV-Based	81	2.0980E2	181.11829	20.12425	0.133	158	0.715
	DTG-Based	79	2.2481E2	209.97357	23.62387			
Viral Load of Patients (copies/mL)	EFV-Based	81	1.3413E5	5.70381E5	63375.69614	3.974	160	0.048*
	DTG-Based	81	3.0494E5	9.70218E5	1.07802E5			
Total Cost of Treatment (Dollars)	EFV-Based	81	7.9248E4	15.84844	1.76094	2.912	160	0.090
	DTG-Based	81	7.9246E4	32.95107	3.66123			

* Significant at *p* < 0.05

### Effectiveness of Antiretroviral Regimens

Using a benchmark of ≤200 copies/mL of viral load as a measure of effectiveness, ART was effective in 58 (71.6 %) of those patients that were initiated on EFV-based regimen, while it was effective in 42 (51.9 %) of patients on DTG-based regimen. Efavirenz-based therapy was thus significantly (p = 0.010) more effective than DTG-based regimen therapy at ≤200 copies/mL. When the viral load benchmark for effectiveness was altered to ≤50, ≤100 and ≤150 copies/mL EFV-based regimen consistently showed higher effectiveness than DTG-based regimen (Table 4)

**Table 4:** Effectiveness of the Antiretroviral Regimens using different Viral Load Benchmarks

Effectiveness of ARV		Type of ARV [n (%)]		Total	Chi-Square	*p*-value

		EFV- Based	DTG- Based			
Viral Load of 50 and less (copies/mL)	Not Effective	40(49.4)	49(60.5)	89(54.9)	2.020	0.155
	Effective	41(50.6)	32(39.5)	73(45.1)		
	Total	81(100.0)	81(100.0)	162(100.0)		
Viral Load of 100 and less (copies/mL)	Not Effective	29(35.8)	43(53.1)	72(44.4)	4.900	0.027*
	Effective	52(64.2)	38(46.9)	90(55.6)		
	Total	81(100.0)	81(100.0)	162(100.0)		
Viral Load of 150 and less (copies/mL)	Not Effective	25(30.9)	43(53.1)	68(42.0)	8.212	0.004*
	Effective	56(69.1)	38(46.9)	94(58.0)		
	Total	81(100.0)	81(100.0)	162(100.0)		
Viral Load of 200 and less (copies/mL)	Not Effective	23(28.4)	39(48.1)	62(38.3)	6.689	0.010*
	Effective	58(71.6)	42(51.9)	100(61.7)		
	Total	81(100.0)	81(100.0)	162(100.0)		

* Significant at *p* < 0.05

### Sociodemographic Predictors of Effectiveness of the ARV Regimen

Multivariate logistic modeling of the effectiveness of the antiretroviral therapy based on sociodemographic characteristics showed that an increase in the duration of disease significantly increased the likelihood of effectiveness at all the viral load benchmarks. At a viral load benchmark of ≤200 copies/mL, a one day increase in the duration of disease increased the likelihood of effectiveness by 1.009 times (100.9%). The model also showed that the odds of treatment effectiveness at ≤50 copies/mL in females compared to males was 0.585 (58.5%). (Supplemental I). When treatment effectiveness was considered for patients on DTG-based regimen alone, the logistic model showed that an increase in the duration of disease significantly increased the likelihood of treatment effectiveness at all the viral load benchmarks. A one-year increase in age also increased the likelihood of DTG being effective by 113.7% at a benchmark of ≤ 200 copies/mL (Supplemental Table II). For those patients on the EFV-based regimen category, no demographic characteristic was a predictor of effectiveness at all viral load benchmarks (Supplemental Table III).

**Table I:** Sociodemographic Predictors of the Effectiveness of the ARV Regimen for All Patients

Characteristics	Effective at VL ≤ 50 copies/mL				Effective at VL ≤ 100 copies/mL				Effective at VL ≤ 150 copies/mL				Effective at VL ≤ 200 copies/mL			

	Exp(B)	95% C.I. for Exp(B)		*p*-value	Exp(B)	95% C.I. for Exp(B)		*p*-value	Exp(B)	95% C.I. for Exp(B)		*p*-value	Exp(B)	95% C.I. for Exp(B)		*p*-value
			
		Lower	Upper			Lower	Upper			Lower	Upper			Lower	Upper	
Duration (days)	1.006	1.002	1.011	.006*	1.009	1.004	1.014	.001*	1.009	1.004	1.014	.001*	1.009	1.004	1.015	.000*
Age (years)	1.035	.987	1.085	.155	1.044	.992	1.099	.097	1.053	.999	1.110	.053	1.032	.977	1.090	.263
Adherence (%)	.997	.989	1.005	.417	.996	.989	1.003	.291	.996	.989	1.003	.259	1.030	.991	1.071	.133
Gender															
Male	Referent															
Female	.585	.244	1.399	.228	.862	.350	2.127	.748	.889	.357	2.218	.802	.989	.392	2.496	.981
Employment Status															
Self Employed	Referent															
Civil Servant	.515	.115	2.312	.387	.649	.143	2.947	.576	.849	.187	3.853	.832	.614	.131	2.886	.537
Unemployed	.479	.093	2.481	.380	.569	.107	3.041	.510	.677	.126	3.638	.649	.633	.110	3.645	.608
Armed Forces	.524	.092	2.971	.465	.617	.103	3.709	.598	.681	.113	4.123	.676	.375	.058	2.417	.303
Student	.462	.067	3.212	.435	1.478	.173	12.634	.721	1.565	.181	13.561	.684	1.043	.117	9.289	.970
Marital Status															
Married	Referent															
Single	.475	.062	3.613	.472	.417	.054	3.226	.402	.425	.055	3.307	.414	.630	.061	6.529	.698
Separated	.255	.033	1.998	.193	.391	.050	3.041	.370	.531	.068	4.138	.546	.639	.061	6.728	.709
Widowed	1.138	.116	11.183	.912	1.500	.134	16.813	.742	1.453	.129	16.349	.762	1.772	.123	25.482	.674
Divorced	.255	.031	2.118	.206	.247	.029	2.083	.198	.226	.027	1.924	.174	.493	.046	5.302	.560

* Significant at *p* < 0.05

**Table II:** Sociodemographic Predictors of the Effectiveness of the ARV for Patients on DTG-based Regimen

Characteristics	Effective at VL ≤ 50 copies/mL				Effective at VL ≤ 100 copies/mL				Effective at VL ≤ 150 copies/mL				Effective at VL ≤ 200 copies/mL			

	Exp(B)	95% C.I. for Exp(B)		*p*-value	Exp(B)	95% C.I. for Exp(B)		*p*-value	Exp(B)	95% C.I. for Exp(B)		*p*-value	Exp(B)	95% C.I. for Exp(B)		*p*-value
			
		Lower	Upper			Lower	Upper			Lower	Upper			Lower	Upper	
Duration (days)	1.014	1.005	1.022	001*	1.018	1.009	1.028	000*	1.018	1.009	1.028	000*	1.021	1.010	1.032	000*
Age (years)	1.097	1.008	1.194	033*	1.098	1.000	1.205	050	1.098	1.000	1.205	050	1.137	1.019	1.269	022*
Adherence (%)	997	987	1.008	631	997	988	1.006	523	997	988	1.006	523	1.033	961	1.109	378
Gender															
Male	Referent															
Female	1.323	275	6.375	727	3.609	665	19.573	137	3.609	665	19.573	137	3.513	598	20.649	164
Employment Status															
Self Employed	Referent															
Civil Servant	271	019	3.885	336	423	027	6.692	541	423	027	6.692	541	053	003	1.048	054
Unemployed	179	010	3.059	235	349	019	6.337	476	349	019	6.337	476	050	002	1.212	066
Armed Forces	991	069	14.310	995	2.299	136	38.847	564	2.299	136	38.847	564	276	015	4.969	383
Student	130	006	2.805	193	1.408	057	34.589	834	1.408	057	34.589	834	251	010	6.420	404
Marital Status															
Married	Referent															
Single	172	011	2.576	202	077	005	1.318	077	077	005	1.318	077	057	002	2.018	115
Separated	057	003	976	048*	058	003	1.039	053	058	003	1.039	053	028	001	1.084	055
Widowed	1.716E9	000		1.000	1.586E9	000		1.000	1.586E9	000		1.000	1.479E8	000		1.000
Divorced	059	002	1.539	089	076	003	2.101	128	076	003	2.101	128	540	016	17.774	730

* Significant at *p* < 0.05

**Table III:** Sociodemographic Predictors of the Effectiveness of the ARV for Patients on EFV-based Regimen

Characteristics	Effective at VL ≤ 50 copies/mL				Effective at VL ≤ 100 copies/mL				Effective at VL ≤ 150 copies/mL				Effective at VL ≤ 200 copies/mL			
	Exp(B)	95% C.I. for Exp(B)		*p*-value	Exp(B)	95% C.I. for Exp(B)		*p*-value	Exp(B)	95% C.I. for Exp(B)		*p*-value	Exp(B)	95% C.I. for Exp(B)		*p*-value
			
		Lower	Upper			Lower	Upper			Lower	Upper			Lower	Upper	
Duration (days)	1.004	.998	1.010	.217	1.006	.998	1.013	.125	1.006	.998	1.014	.120	1.007	.998	1.015	.130
Age (years)	.996	.932	1.064	.907	1.008	.937	1.085	.827	1.021	.942	1.106	.615	.976	.896	1.062	.571
Adherence (%)	1.011	.964	1.061	.640	1.024	.973	1.079	.362	1.050	.991	1.113	.098	1.058	.996	1.123	.067
Gender															
Male	Referent															
Female	.530	.143	1.964	.342	.487	.109	2.172	.346	.456	.083	2.499	.365	.775	.136	4.427	.775
Employment Status															
Self Employed	Referent															
Civil Servant	.284	.025	3.241	.311	.000	.000	.	.999	.000	.000	.	.999	.000	.000	.	.999
Unemployed	.280	.019	4.044	.350	.000	.000	.	.999	.000	.000	.	.999	.000	.000	.	.999
Armed Forces	.117	.005	2.933	.192	.000	.000	.	.999	.000	.000	.	.999	.000	.000	.	.999
Student	5.041E8	.000	.	.999	1.042	.000	.	1.000	1.297	.000	.	1.000	1.192	.000	.	1.000
Marital Status															
Married	Referent															
Single	.000	.000	.	1.000	.000	.000	.	1.000	0.000	0.000	.	1.000	.000	.000	.	1.000
Separated	.000	.000	.	1.000	.000	.000	.	1.000	0.000	0.000	.	1.000	.000	.000	.	1.000
Widowed	.000	.000	.	1.000	.000	.000	.	1.000	0.000	0.000	.	1.000	.000	.000	.	1.000
Divorced	.000	.000	.	1.000	.000	.000	.	1.000	0.000	0.000	.	1.000	.000	.000	.	1.000

* Significant at *p* < 0.05

### Cost-effectiveness

The total cost of treatment for patients on the EFV-based regimen was $6,419,121.12, which translated to an average cost of $79,248.41 per patient. The average cost-effectiveness of EFV-based regimen at a viral load of ≤200 copies/mL in dollar per effectiveness($/effectiveness) was $110,682.14. For patients on DTG-based regimen, the average cost of treatment and average cost effectiveness (ACE) at 200 copies/mL were $79,246.34 and $152,690.44 respectively. Meanwhile, the incremental cost-effective ratio (ICER) of patients on DTG compared to those on EFV at a viral load of 200 copies/mL was $10.5076 per effectiveness. The cost-effectiveness analysis is presented in Table 5.

**Table 5:** Cost-Effectiveness Analysis between DTG and EFV Regimens

Characteristics	Type of Regimen	

	EFV-Based	DTG-Based
Frequency	81	81
Total Cost ($)	6419121.12	6418953.72
Average Cost ($) Per Patient	79248.41	79246.34
Effectiveness at ≤250copies/mL (%)	50.6	39.5
Effectiveness at ≤100 copies/mL (%)	64.2	46.9
Effectiveness at ≤150 copies/mL (%)	69.1	46.9
Effectiveness at ≤200 copies/mL (%)	71.6	51.9
ACE at ≤50 copies/mL ($/effectiveness)	156617.41	200623.65
ACE at ≤100 copies/mL ($/effectiveness)	123439.89	168968.74
ACE at ≤150 copies/mL ($/effectiveness)	114686.56	168968.74
ACE at ≤200 copies/mL ($/effectiveness)	110682.14	152690.44
ICER at ≤50 copies/mL ($/effectiveness)	18.6486
ICER at ≤100 copies/mL ($/effectiveness)	11.9653
ICER at ≤150 copies/mL ($/effectiveness)	9.3243
ICER at ≤200 copies/mL ($/effectiveness)	10.5076

ACE: Average Cost-Effectiveness Ratio; ICER: Incremental Cost-Effectiveness Ratio

### Sensitivity Analysis

The benchmark for treatment effectiveness was varied to 150, 100, 50 copies/mL and the ICER was determined at these viral load points. The result (Table 5) showed an increase in the ICER at a viral load benchmark of 100 and 50 copies/mL from the base case scenario. The ICER, however, decreased to $9.3243at a viral load benchmark of 150 copies/mL.

## Discussion

This study explored the sociodemographic and clinical characteristics of HIV-positive patients in a hospital in Nigeria. It also evaluated the cost-effectiveness of DTG compared to EFV-based regimens in the treatment naïve patients.

Majority of the patients in the study fell within the age category of 20 to 49 years. This indicates that new HIV-infection is still most prevalent among adults that are within their most productive years. This is further reflected in the marital and employment status of the respondents where most of them were married and were gainfully employed. These findings are consistent with the demographic characteristics of HIV patients in Nigeria, as reported by several other studies 19,20. Females formed the highest proportion of patients in the study. Females generally constitute the majority of the population of HIV patients in Nigeria 21 possibly because of the social and cultural characteristics that make women more vulnerable to HIV 1.

The mean CD4 count in this study was such that more than a quarter of patients in both categories of regimens reporting a CD4 count of less than 200 cells/uL. The mean CD4 count was similar to those abstained in a cohort of patients after 6 months of initiating ART 22. In contrast to the CD4 count, the viral load test result was, however, less than the viral suppression range of 200 copies/mL in majority of the patients from both categories. Although a baseline viral load was not available from this study, patients initiating ART usually have a baseline viral load of more than 1000 copies/mL 23. It could therefore be said that the viral load of patients in our study declined rapidly in the early stage of treatment initiation. Rapid reduction in viral load is observed in other studies involving treatment-naïve patients initiating ART, especially when initiated early after diagnosis 23–25.

At the viral load threshold of 200 copies/mL and less, EFV-based regimen showed significantly higher effectiveness than DTG. This finding contradicts that of several other studies that assessed the efficacy of DTG and EFV-based regimens in treatment-naïve patients 26–28. The SINGLE trial showed DTG-based regimen to have a better viral suppression rate than EFV-based regimen (88% vs 81%), making it more effective than EFV-based regimen (26). In another related meta-analysis, the odds of DTG being more effective than the EFV-based regimen was 1.86 (C. I= 1.40-2.47) 28. Although these studies used a lower benchmark for viral suppression and time duration of 48 weeks, our findings are still comparable with these studies. We found that even when the viral load threshold was lowered to 150, 100, and 50 copies/mL, EFV consistently maintained a higher level of effectiveness than the DTG-based regimen.

We found from our results, some sociodemographic predictors of antiretroviral effectiveness in the cohort of patients in this study, regardless of the ART used. The duration of disease was a significant predictor of treatment effectiveness at 200 copies/mL and lower thresholds. The duration of disease roughly equates to the duration of treatment in the test and treat model of HIV treatment. A longer duration of disease, therefore, indicates a longer treatment duration which confers a higher chance of viral suppression 23, 29.

The economic analysis showed that the average cost of treatment per patient was slightly higher for the EFV than DTG-based regimen. EFV-based regimen also demonstrated a lower cost per effectiveness than DTG, at ≤200 copies/mL. This is largely attributed to the higher effectiveness shown by EFV-based regimen on the current viral load test. A comparison of DTG and EFV-based regimens showed a positive ICER/effectiveness at a viral load benchmark of ≤200 copies/mL. DTG-based regimen was therefore considered a cost-effective strategy compared to EFV-based regimen because the ICER was lower than 1% of the Nigerian per capita GDP for 2019. Our cost-effectiveness findings are similar to that of several other studies that evaluated the cost-effectiveness of DTG and EFV regimens, either with Tenofovir/Lamivudine (TDF/3TC) backbone 30,31 or other backbones 32. One of such studies was that conducted to determine the cost-effectiveness of the DTG-based regimen in India. The study found that the lifetime incremental cost-effectiveness ratio of DTG compared to EFV was $130/Years of Life Saved (YLS), which was less than 10% of the Indian 2015 per capita GDP 30. The cost-effectiveness of DTG was also demonstrated in a study among HIV-1 patients in Canada. The Dolutegravir-based regimen was found to be dominant over the EFV-based regimen (more clinically effective and cost-saving) in both treatment naïve and treatment-experienced patients with a lifetime cost saving of Can$ 7735 32.

The results of Peng et al, was however, contrary to our findings. Their study showed that DTG-based regimen was not cost-effective when compared to EFV-based regimen. The clinical outcomes of DTG were found to be similar to that of EFV-based regimen, with DTG resulting in a marginal increase in QALY of +0.12 over a lifetime horizon. Also, the lifetime cost of Dolutegravir/Tenofovir/Emtricitabine (DTG/TDF/FTC) was $19,153 higher than that of Efavirenz/Tenofovir/Emtricitabine (EFV/TDF/FT). The ICER was $272,389, which was higher than the traditional US willingness to pay threshold of $50,000/QALY gained in all scenarios 12.

This study has some limitations. First, we only considered the cost-effectiveness of the two ARTs over a short-term horizon (less than a year for most patients). Our study did not extrapolate the ICER to the medium term, and over the lifetime horizon. Evidence from other studies showed that the cost-effectiveness of DTG over EFV-based regimen progressively increases in the long term 31 possibility because the clinical benefit of DTG mostly accrued in the later stage of treatment 32. A future study that models the cost-effectiveness of DTG over a lifetime horizon will provide a more detailed picture of its cost-effectiveness and a better basis for decision making. Second, we used the CDC benchmark for viral suppression (<200 copies/mL) as the surrogate for treatment effectiveness. The Nigerian HIV treatment guideline uses a higher benchmark of ≤1000 copies/mL to determine viral suppression^33^. The use of a more conservative benchmark in this study may have resulted in an overestimation of the ICER result. Lastly, we did not consider the cost of treating adverse effects associated with the use of each of the competing alternatives. Adverse effects occur disproportionately in the two ART groups, with EFV showing a higher rate of adverse effects than DTG 26. The incorporation of the cost of treating adverse effects would have resulted in a lower cost of treatment for DTG compared to EFV-based regimen.

## Conclusion

Efavirenz based regimen had higher treatment effectiveness than DTG-based regimen in treatment-naive patients after initiating treatment in a short term. Socio-demographic variables such as duration of disease and gender significantly predict treatment effectiveness. Compared to EFV, DTG-based regimen is cost-effective in the management of treatment naïve HIV patients, in the early phase of treatment, from the provider's perspective in Nigeria.
